# Associations between couples’ work–life balance and pregnant women’s quality of life in Japan: a cross-sectional dyadic study

**DOI:** 10.1186/s12884-026-09733-7

**Published:** 2026-07-28

**Authors:** Aoi Makino, Shigemi Iriyama

**Affiliations:** 1 Department of Integrated Health Sciences, Nagoya University Graduate School of Medicine, Nagoya University, Nagoya, Japan; 2 Faculty of Nursing and Social Welfare Sciences, Fukui Prefectural University, Fukui, Japan

**Keywords:** Work–life balance, Quality of life, Marital satisfaction, Pregnancy, Dual-income couples

## Abstract

**Background:**

In dual-income couples, maintaining pregnant women’s quality of life (QOL) while achieving work–life balance (WLB) is essential. This study examined the association between WLB and pregnant women’s QOL among dual-income couples at different stages of pregnancy.

**Methods:**

This cross-sectional, dyadic study included pregnant women and their husbands (*N* = 150 couples). Paired questionnaires were distributed in cooperation with obstetric facilities in Japan. Participants were categorized into the first, second, and third trimesters for stratified analysis. WLB was assessed using the Japanese version of the Survey Work–Home Interaction–Nijmegen (SWING-J), which comprises four subscales: work-to-family negative spillover (WFNS), family-to-work negative spillover, work-to-family positive spillover, and family-to-work positive spillover (FWPS). QOL was measured using the WHOQOL-BREF. Multivariate logistic regression analyses were performed after adjusting for marital relationship quality (Quality Marriage Index).

**Results:**

No significant association was observed between WLB and QOL in the first and second trimesters. In the third trimester, higher WFNS among pregnant women was significantly associated with lower QOL (aOR = 0.179, 95% CI = 0.038–0.832). Regarding husbands’ WLB, higher WFNS was positively associated with pregnant women’s QOL (aOR = 11.168, 95% CI = 1.443–86.419), whereas higher FWPS was negatively associated with pregnant women’s QOL (aOR = 0.063, 95% CI = 0.007–0.554).

**Conclusion:**

In the third trimester, pregnant women’s QOL was associated with both negative and positive aspects of WLB at the couple level. Higher WFNS among pregnant women and higher FWPS among husbands were associated with a lower QOL, whereas higher WFNS was associated with a higher QOL. These findings suggest that multiple WLB-related factors, both negative and positive, are linked to pregnant women’s QOL, highlighting the importance of considering complex spillover dynamics when supporting maternal well-being in dual-income households.

## Background

Pregnant women are vulnerable to physical and psychological instability, and their quality of life (QOL) during pregnancy has been shown to significantly influence postpartum mental health [1, 2]. Therefore, maintaining and improving their QOL throughout the gestational period is critical for maternal health.

Even among women with uncomplicated pregnancies, maintaining their QOL remains challenging. Physical QOL can decline due to pregnancy-related symptoms such as morning sickness in early pregnancy and lower back pain in the later stages [3]. A longitudinal study of pregnant Japanese women demonstrated that the physical QOL decreased in the first trimester, whereas the psychological QOL temporarily improved in the second trimester; nevertheless, the overall QOL tended to decline across pregnancy [4].

In Japan, the number of dual-income households has been increasing annually, with a growing proportion of couples working full-time [5]. Various child-rearing support policies have been implemented to promote work–life balance (WLB), including those targeting fathers. Paternal role acquisition is facilitated by cooperative marital associations and joint involvement during pregnancy [6]. However, husbands with longer working hours are more likely to exhibit depressive tendencies, which may hinder WLB maintenance [7]. Although collaborative efforts to sustain WLB during pregnancy are desirable, they are often difficult to achieve.

WLB comprises negative spillover (NS) and positive spillover (PS), both of which operate bidirectionally between the work and family domains [8]. NS refers to role conflicts arising from competing work and family demands, whereas PS reflects the synergistic benefits of engaging in multiple roles. NS has been associated with adverse health outcomes, as explained by occupational stress models, such as the National Institute for Occupational Safety and Health (NIOSH) framework [9]. In contrast, PS has been linked to a better QOL and fewer depressive symptoms [10]. Importantly, NS and PS may mutually influence each other within couples, resulting in crossover effects whereby one partner’s experiences affect the other’s well-being [11].

Although several studies on WLB have focused on couples in the child-rearing stage [12], research examining WLB during pregnancy remains limited. Although WLB is considered an important factor related to QOL in pregnant women, evidence of an association between WLB and QOL during pregnancy is scarce. To the best of our knowledge, no study has specifically investigated how different aspects of WLB in dual-income couples are associated with pregnant women’s QOL.

Therefore, this study aimed to clarify the association between WLB and QOL during pregnancy in Japanese dual-income couples. These findings are expected to contribute to improvements in nursing care and inform interventions targeting couples during the prenatal stage. We hypothesized that (1) pregnant women with higher PS would report higher QOL; (2) pregnant women with lower NS would report higher QOL; (3) pregnant women whose husbands had higher PS would report higher QOL; and (4) pregnant women whose husbands had lower NS would report higher QOL.

## Methods

### Study setting and participants

A convenience sampling approach was adopted to recruit participants from cooperative obstetric care facilities. Seven facilities comprising two university hospitals and five obstetric clinics agreed to participate in the study. These facilities were located across Aomori, Fukui, Aichi, and Osaka prefectures. Staff at these facilities distributed paired questionnaires to eligible participants attending prenatal checkups or parent education classes. The purpose and procedures of the study were explained to the pregnant women and their husbands, both verbally and in writing. If the husband was absent, the pregnant woman was asked to provide him with the corresponding questionnaire at her convenience. Each pair of questionnaires was assigned a unique identification number to link the husbands’ and wives’ responses. To ensure confidentiality and avoid mutual influence, completed questionnaires were returned separately by mail. The data were collected between August 2023 and December 2024.

All eligible participants were dual-income couples. Couples in which either the partner was unemployed or younger than 20 years were excluded.

The required sample size was estimated using G*Power 3.1. Assuming an effect size of 0.15, a significance level of 0.05, a statistical power of 0.80, and 10 independent variables for binary logistic regression multiple analysis, the minimum required sample size was calculated to be 118. In the present study, a total of 150 couples were included in the analysis.

### Data collection and measures

Data were collected using self-administered questionnaires to assess the sociodemographic characteristics, employment environment, marital satisfaction, WLB, and pregnant women’s QOL.

### Socio-demographic characteristics

The socio-demographic variables included age, planned pregnancy (yes/no), involvement in household chores, and counselor availability. For pregnant women, the gestational age, parity, and family composition were also assessed.

### Employment environment

Employment-related variables included pregnancy-related job reassignment, employment status (full-time/part-time), executive roles, night shifts, and overtime work. Workplace support was assessed using items related to pregnancy-related consultations and availability of leave. Workplace safety considerations during pregnancy were also assessed.

### Marital satisfaction

Marital satisfaction was conceptualized as a global subjective evaluation of the quality of one’s marital relationship following Norton’s definition [13]. Marital satisfaction was measured using the Japanese version of the Quality Marriage Index (QMI) originally developed by Norton and adapted by Moroi [14]. The QMI comprises six items rated on a four-point Likert scale (1–4), yielding total scores ranging from 6 to 24, with higher scores indicating greater marital satisfaction. Internal consistency was high for both pregnant women (Cronbach’s α = 0.927) and husbands (Cronbach’s α = 0.920).

### Work–life balance

Following Greenhaus and Allen [15], WLB was conceptualized as an individual’s ability to meet the demands of both work and personal life while experiencing satisfaction and effective functioning in both domains. WLB was assessed using the Japanese version of the Survey Work–Home Interaction-Nijmegen (SWING-J) [16]. SWING-J comprises 22 items across four subscales: work-to-family negative spillover (WFNS; eight items), family-to-work negative spillover (FWNS; four items), work-to-family positive spillover (WFPS; five items), and family-to-work positive spillover (FWPS; five items). Items were rated on a four-point Likert scale (0–3), with higher scores indicating stronger spillover effects. Internal consistency ranged from acceptable to high (Cronbach’s α = 0.716–0.865 for pregnant women and 0.780–0.865 for husbands).

### Quality of life

The QOL of the pregnant women was measured using the Japanese version of the World Health Organization Quality of Life (WHOQOL-BREF) [17]. This instrument comprises 26 items assessing four domains (physical, psychological, social, and environmental), as well as the overall QOL. The items were rated on a five-point Likert scale. Domain and overall QOL scores were calculated according to the WHO Health Organization scoring guidelines, with higher scores indicating better QOL. Cronbach’s α values were 0.79 for the physical domain, 0.81 for the psychological domain, 0.68 for the social domain, and 0.83 for the environmental domain.

### Data analysis

The participants were divided into two independent groups according to gestational stage: the first and second trimester (< 28 weeks of gestation) and the third trimester (≥ 28 weeks of gestation). Differences in the QMI, SWING-J subscale scores, and pregnant women’s QOL between gestational stages were examined using the Mann–Whitney U test, as the data were not normally distributed. For the regression analyses, the QMI and each SWING-J subscale score were dichotomized into high and low groups based on the median values.

Binary logistic regression analyses were conducted separately for the first and second trimester and third trimester groups, with QOL as the dependent variable. The independent variables included pregnant women’s and husbands’ SWING-J subscale scores, and the QMI scores for both partners were included as covariates to adjust for marital relationship quality. Marital relationship quality was included as a covariate because of its well-established association with both WLB and QOL [18]. Other participant characteristics were also examined as potential covariates; however, none revealed a significant association with pregnant women’s QOL. Furthermore, to prevent the risk of overfitting and unstable estimates within the relatively limited sample size in the stratified analyses, no additional covariates were introduced.

Statistical analyses were performed using SPSS version 28.0 (IBM Corp., Armonk, NY, USA). Statistical significance was set at *p* < 0.05 (two-tailed).

## Results

Questionnaires were administered to 680 couples. Paired responses were obtained from 198 couples, of which 150 couples with complete and valid data were included in the final analysis (collection rate: 29.1%, valid response rate: 75.8%).

The median age of the wives was 32.2 years (IQR: 29.0–35.3) and that of their husbands was 33.0 years (IQR: 30.0–37.0). The median gestational age was 27.5 weeks (IQR: 17.0–34.3). Overall, 60.0% of the participants were primiparous and 90.0% lived in nuclear families. Most couples (92.6%) reported planned pregnancies, 46.0% reported receiving support from household chores, and 78.0% reported having someone they trusted (Table 1).

**Table 1.**
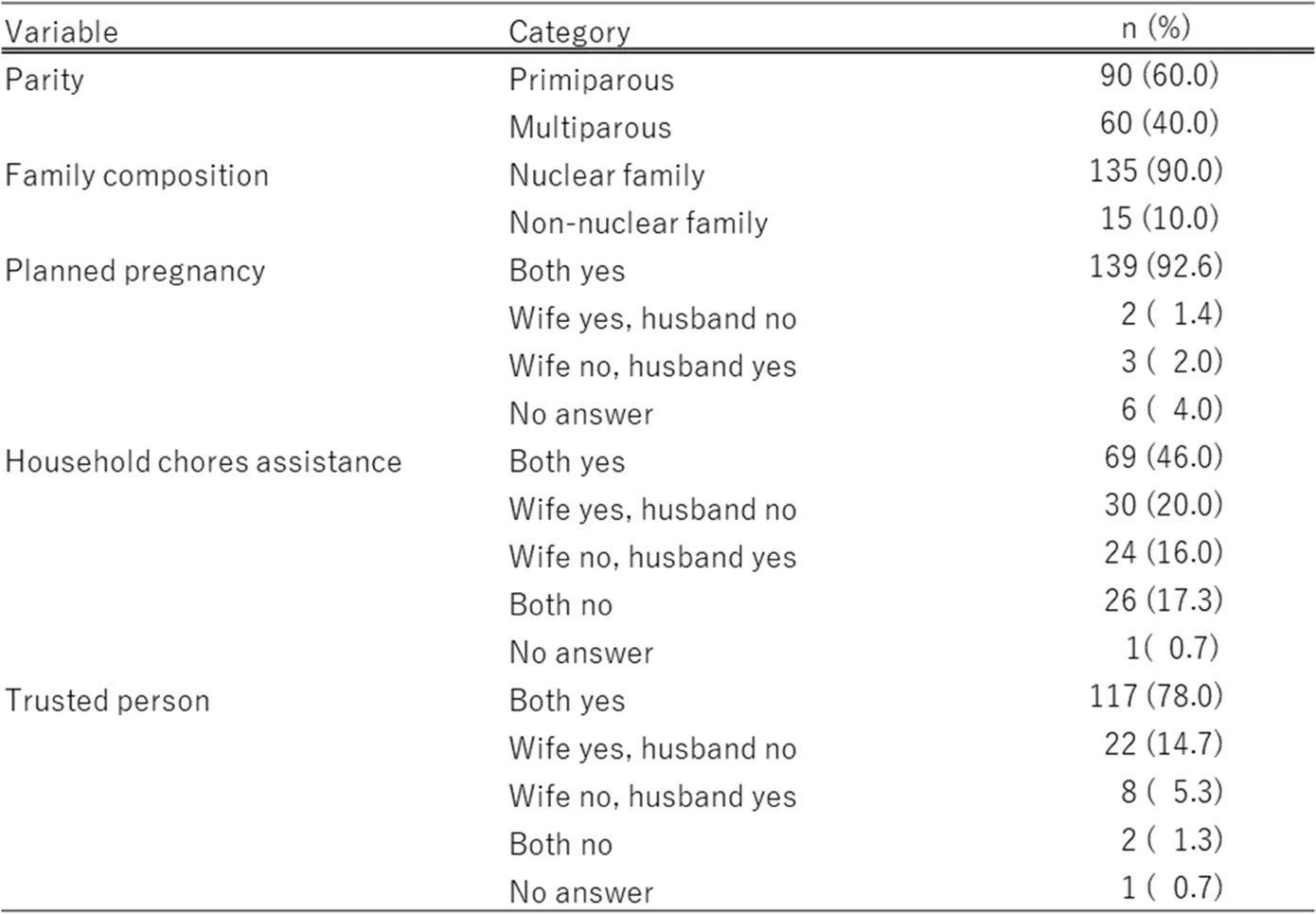
Socio-demographic characteristics of participating couples (*N* = 150 couple)

Regarding employment characteristics, 26.7% of the wives and 9.3% of the husbands reported pregnancy-related job reassignment. Full-time employment was more common among husbands than among wives (93.3% vs. 77.3%), whereas part-time employment was more frequent among wives (22.0% vs. 6.7%). The husbands also held executive roles more frequently (20.0% vs. 7.4%), worked night shifts (28.0% vs. 5.3%), and engaged in overtime work (74.0% vs. 39.3%). Although a small proportion of wives reported night shifts and overtime work, these practices were legally exempt upon request. Supportive workplace attitudes toward pregnancy were reported by 90.0% of wives and 93.3% of husbands. Workplace mentoring was reported by 78.7% of both groups. Requests for workplace accommodations were accepted for 84.0% of wives and 72.7% of husbands. Additionally, 86.7% of the wives reported receiving maternal safety considerations at their workplaces (Table 2).

**Table 2.**
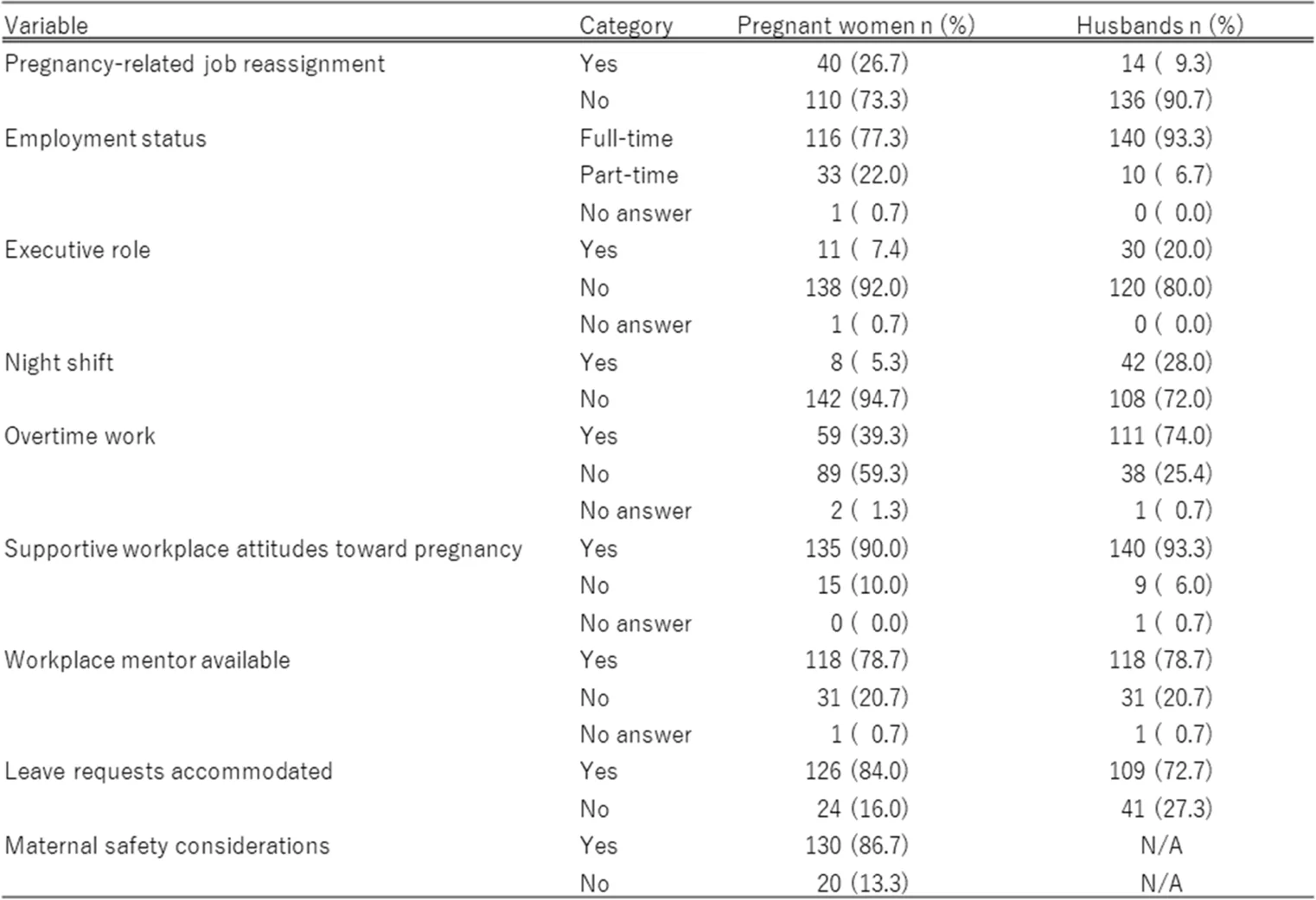
Employment  characteristics of participating couples (*N* = 150 couples)

The QMI, SWING-J subscales, and QOL were compared between the pregnant women in the first and second trimesters and those in the third trimester. The median QMI score was 22.00 (IQR: 18.00–24.00) in the first and second trimesters and 21.00 (18.00–24.00) in the third trimester, with no significant difference between the groups (*p* = 0.879). The median WFNS score was 2.00 (1.00–5.00) and 3.00 (1.00–7.00), respectively (*p* = 0.850), and the FWNS score was 0.00 (0.00–0.00) and 0.00 (0.00–1.00) (*p* = 0.397). In contrast, the positive spillover scores were significantly higher in the third trimester. WFPS increased from 7.00 (3.00–9.00) to 8.00 (6.00–10.00) (*p* = 0.004), and FWPS increased from 8.00 (4.00–10.00) to 9.00 (6.00–10.00) (*p* = 0.036). The QOL scores did not differ significantly between the groups (3.30 [3.19–3.88] vs. 3.54 [3.23–3.88], *p* = 0.482).

For husbands, the QMI and SWING-J scores were compared between the same gestational groups. The median QMI score was 22.00 (18.00–24.00) in the first and second trimesters and 23.00 (20.00–24.00) in the third trimester (*p* = 0.243). No significant differences were observed in any SWING-J subscale: WFNS (6.00 [3.00–8.00] vs. 5.00 [3.00–8.00], *p* = 0.907), FWNS (0.00 [0.00–2.00] vs. 0.00 [0.00–3.00], *p* = 0.845), WFPS (7.00 [5.00–9.00] vs. 8.00 [5.00–10.00], *p* = 0.281), and FWPS (7.00 [5.00–10.00] vs. 8.00 [5.00–10.00], *p* = 0.375).

Table 3 shows the logistic regression analysis examining the association between pregnant women’s average QOL and couples’ WLB in the first and second trimesters, adjusted for QMI. None of the SWING-J subscales for wives (WFNS, FWNS, WFPS, and FWPS) or husbands were significantly associated with pregnant women’s QOL in either the crude or adjusted models.

**Table 3.**
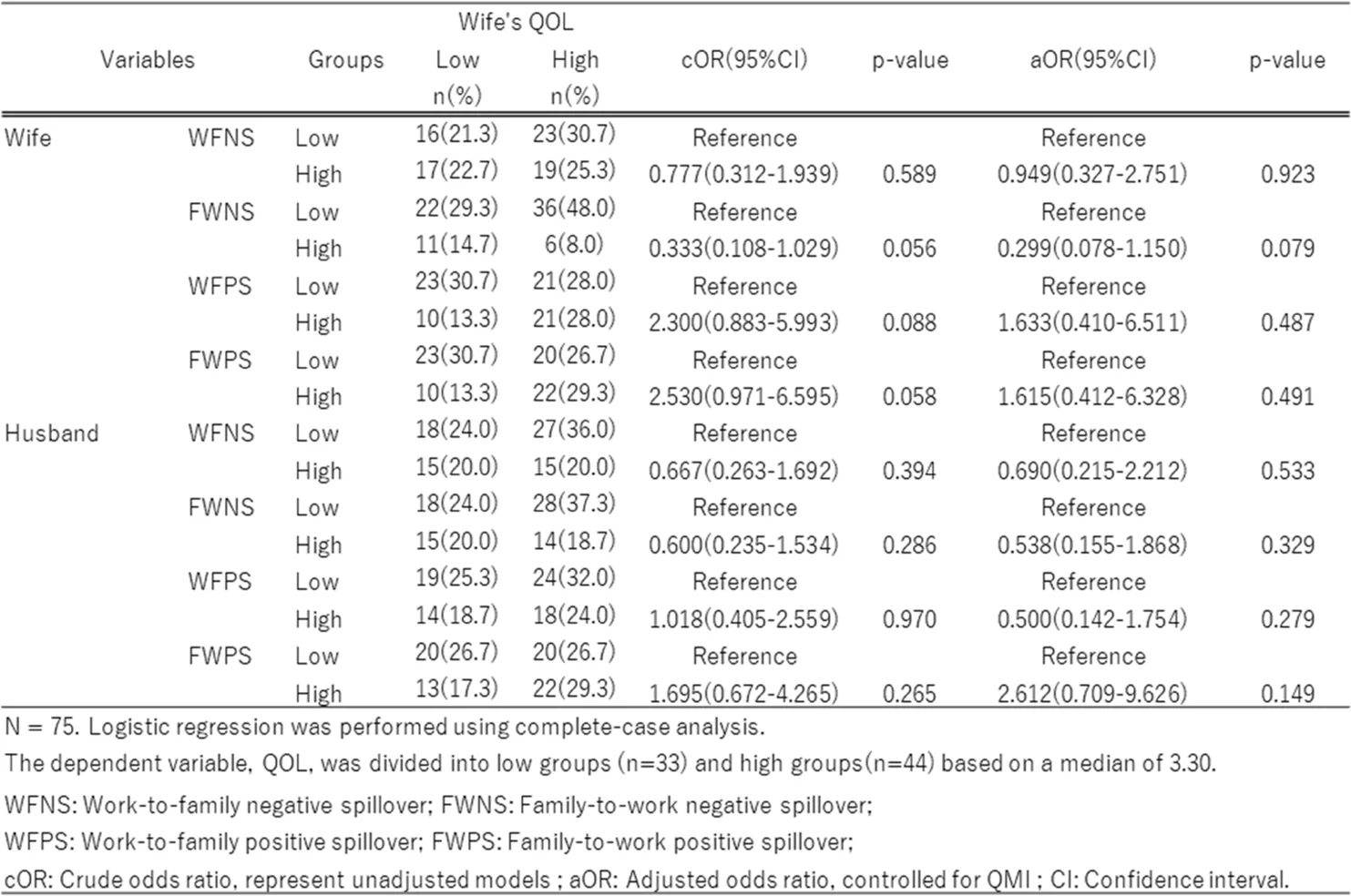
Logistic regression analysis of associations between couples’ WLB and pregnant women’s QOL in the first and second trimesters

Table 4 presents the logistic regression analysis of the associations between pregnant women’s average QOL and couples’ WLB during the third trimester. Among the wives, higher WFNS scores were significantly associated with lower QOL in both the crude and adjusted models (cOR = 0.196, 95% CI: 0.073–0.522, *p* = 0.001; aOR = 0.179, 95% CI: 0.038–0.832, *p* = 0.028). Higher FWNS scores were also associated with lower QOL in the crude model (cOR = 0.164, 95% CI: 0.052–0.516, *p* = 0.002), although this association was not significant after adjusting for QMI (*p* = 0.052). No significant associations were observed between the wives’ WFPS or FWPS scores.

**Table 4.**
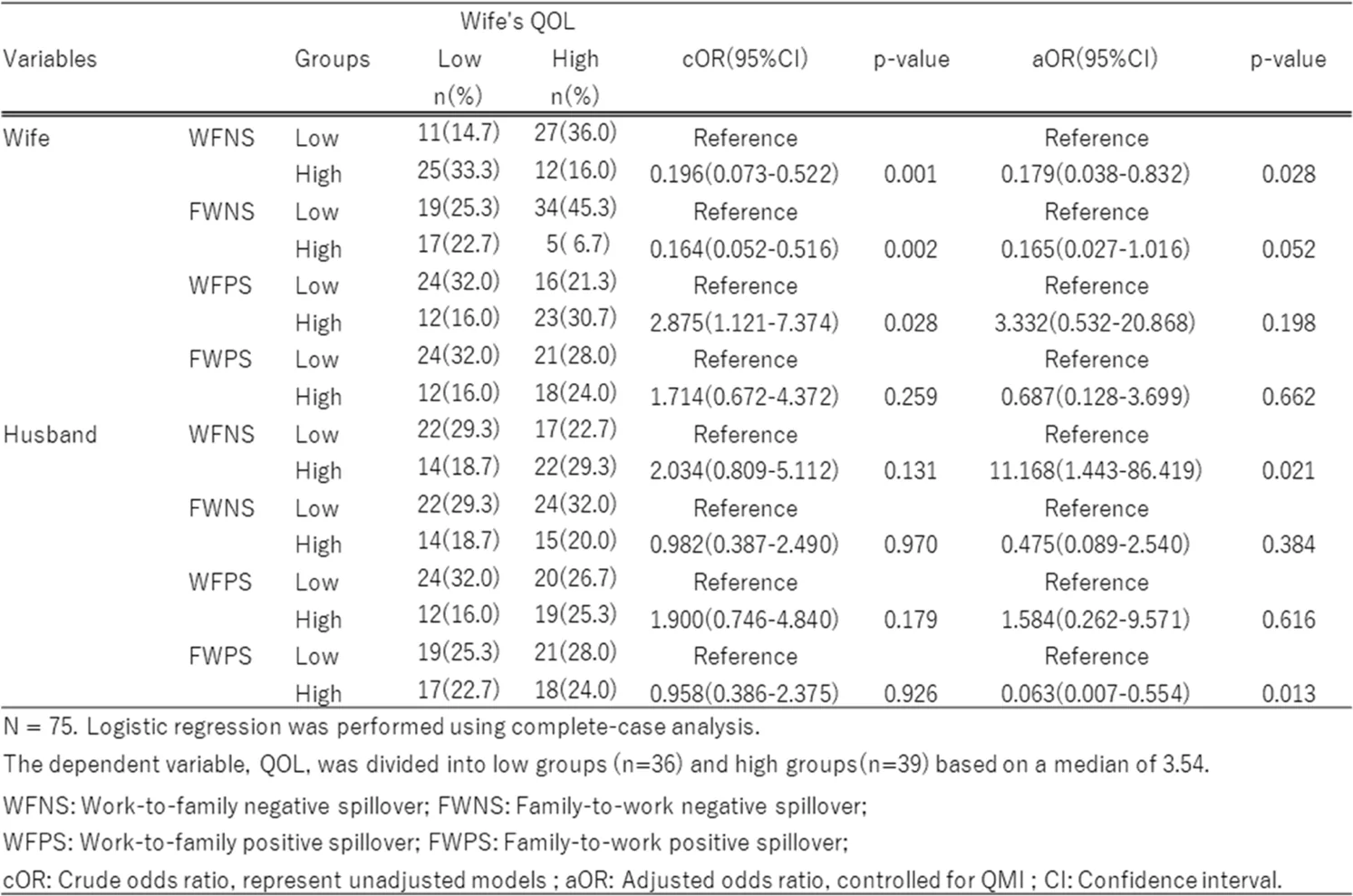
Logistic regression analysis of associations between couples’ WLB and pregnant women’s QOL in the third trimesters

Among the husbands, higher WFNS scores were associated with higher pregnant women’s QOL in the adjusted model (aOR = 11.168, 95% CI: 1.443–86.419, *p* = 0.021), whereas higher FWPS scores were significantly associated with lower pregnant women’s QOL (aOR = 0.063, 95% CI: 0.007–0.554, *p* = 0.013). No significant associations were observed between the husbands’ FWNS or WFPS scores.

## Discussion

This study examined the association between WLB and pregnant women’s QOL among dual-income couples. Specifically, we analyzed the associations between both partners’ WLB and pregnant women’s QOL across different stages of pregnancy. The results showed that both pregnant women’ and husbands’ WLB were significantly associated with QOL in the third trimester, whereas no such association was observed in the first or second trimesters (Tables 3 and 4). These findings suggest that sensitivity to WLB-related stress may increase as pregnancy progresses, likely due to the intensifying physical and psychological demands of childbirth.

Consistent with our hypotheses, higher levels of negative spillover (NS) among pregnant women were associated with lower QOL in the third trimester. Both work-to-family and family-to-work NS were negatively associated with QOL, which is consistent with previous research on dual-income couples with infants [19]. Although the level of WFNS observed in this study was lower than that reported in couples with preschool-aged children [18], the detrimental impact of both spillover directions underscores the importance of addressing WLB before childbirth.

WFNS occurs when work demands interfere with family life. Previous studies have identified high work demands as antecedents of NS, whereas workplace resources, such as supportive attitudes, act as buffers [20]. In this study, most pregnant women reported access to workplace resources, including supportive attitudes (90.0%) and mentoring (78.7%) (Table 2). Nevertheless, a notable proportion was still engaged in overtime work or night shifts, and 13.3% indicated that their maternal safety was not adequately protected. Concerns regarding fetal safety and feelings of guilt when balancing work and pregnancy may exacerbate WFNS, negatively affecting QOL [21]. These findings highlight the need to reduce objective work burdens, particularly in the third trimester, regardless of the perceived “supportiveness” of the environment.

Findings regarding husbands’ WLB are partially inconsistent with our hypotheses. Contrary to expectations, higher FWPS among husbands was associated with lower pregnant women’s QOL in the third trimester. FWPS reflects motivation gained from family roles, which enhances work engagement [22]. In the context of the long working hours common among Japanese men [23], greater FWPS may prompt the husbands to increase their work engagement, potentially at the expense of the time spent providing direct support at home. This imbalance may reduce the emotional or practical support available to pregnant women, thereby affecting their QOL.

In contrast, higher levels of husbands’ WFNS were positively associated with pregnant women’s QOL. This unexpected finding (aOR = 11.168; Table 4), together with the wide confidence interval, should be interpreted with caution. The husbands reported significant work burdens, including managerial roles and night shifts. Higher WFNS may reflect a husband’s greater awareness of the impact of his work on his family [24], which could trigger compensatory behaviors or increase emotional sensitivity toward his partner. Furthermore, despite 90.0% of participants lived in nuclear families, the study population reported relatively high levels of perceived social support; 66.0% of wives received assistance with household chores and 92.7% had a trusted person to consult. Such a supportive environment may have buffered the potential negative effects of husbands’ work-related strain. While the present study did not directly examine this mechanism, these alternative pathways may partly explain the observed associations. This aligns with previous research suggesting that social capital and social support are important determinants of maternal health during pregnancy [25]. Moreover, notably, while wives’ QMI was a strong predictor of their QOL, WLB factors remained significant even after adjusting for QMI, indicating that WLB affects QOL through pathways independent of marital satisfaction.

This study has several limitations that should be acknowledged.

First, convenience sampling limits the generalizability of the findings. Because the participants were recruited from cooperating facilities rather than through probability sampling, the results may not be fully representative of all dual-income couples in Japan.

Second, questionnaires for husbands were distributed and explained to pregnant women. This recruitment method may have introduced a selection bias, favoring couples with relatively strong marital associations and greater mutual awareness of each other’s work situations. Consistent with this possibility, marital associations in the present sample were generally positive, and marital satisfaction emerged as a factor with a strong correlation with pregnant women’s QOL. Therefore, the observed associations between WLB and QOL may not fully represent couples experiencing greater relational strain.

Third, data were collected from facilities in four prefectures (Aomori, Fukui, Aichi, and Osaka). Although these sites represent geographically diverse regions of Japan, regional differences in cultural norms, employment environments, and healthcare systems were not controlled. Therefore, unmeasured regional factors may have influenced the findings, and generalizability to all regions of Japan may be limited.

Fourth, the cross-sectional design precludes causal inferences regarding the association between couples’ WLB and pregnant women’s QOL. Additionally, this study focused exclusively on pregnant women’s QOL and did not assess their husbands’ QOL, thereby limiting its ability to examine reciprocal or dyadic effects. Future studies should employ longitudinal designs with larger samples and include QOL assessments of both partners to comprehensively elucidate the dynamic association between WLB and QOL among couples during pregnancy.

Fifth, although the overall sample size met the required number estimated by the power analysis, the stratified analyses resulted in smaller subgroup sizes. This reduction may have diminished statistical power, particularly for the regression analyses conducted separately across gestational stage. Therefore, these findings should be interpreted with caution, and further studies utilizing larger samples are warranted.

## Conclusion

The pregnant women’s QOL was significantly lower when their NS was high, highlighting the importance of reducing work-related burdens that interfere with family life. Additionally, pregnant women’s QOL declined when their husbands exhibited high FWPS scores, suggesting that excessive work engagement among husbands may limit their ability to provide adequate support during pregnancy. Conversely, higher WFNS among husbands was positively associated with pregnant women’s QOL. However, this unexpected finding should be interpreted with caution, as it may reflect complex spillover dynamics within couples.

Taken together, these findings indicate that both negative and positive aspects of WLB within couples contribute to pregnant women’s QOL, emphasizing the need to consider the interactive and dynamic nature of spillover processes when supporting maternal well-being.

## Data Availability

The datasets used and analysed during the current study are available from the corresponding author on reasonable request.
